# 116 Clinical Impact of Transitioning from a Burn Provider-based to Hospitalist-based Sedation Service

**DOI:** 10.1093/jbcr/irac012.118

**Published:** 2022-03-23

**Authors:** Anjay Khandelwal, Jeffrey D Solomon, Beverly Beaucock, Richard B Lou

**Affiliations:** Akron Dallas's Hospital, Akron, Ohio; Akron Dallas's Hospital, Akron, Ohio; Akron Dallas's Hospital, Akron, Ohio; Akron Dallas's Hospital, Akron, Ohio

## Abstract

**Introduction:**

Burn patients often experience a tremendous amount of pain and anxiety during dressing changes and other procedures, frequently requiring a moderate sedation (MS) or deep sedation (DS) for successful completion. We previously reported our primarily Nurse Practitioner-based model for procedural sedation but recently transitioned to a primarily hospitalist-based sedation service. We evaluated the clinical and financial impact of this transition.

**Methods:**

Retrospective chart review of patients undergoing MS or DS from June 2019 to June 2020 (burn provider-based [BPB]) and August 2020 to August 2021 (hospitalist-based [HB]). Data included demographics, number and types of sedation, provider type, American Society of Anesthesiology Physical Status Classification (ASA score), complications and relative value units (RVU)/anesthesia units generated. Our hospitalist completed a combined Internal Medicine-Pediatrics residency and is credentialed by the institution for procedural sedation.

**Results:**

During the BPB sedation timeframe, 263 patients were admitted to the burn center, of which 55 patients (21%) underwent 203 sedations (average: 3.6 sedations/patient). Twenty-one (10%) were DS. The most common medications used for DS were a combination of Midazolam, Ketamine, Fentanyl, Glycopyrrolate and Ondansetron (81%); and for MS, a combination of Fentanyl and Midazolam (77.5%). Ten percent of patients had an ASA of 3 or greater.

During the HB sedation timeframe, 203 patients were admitted, of which 73 patients (36%) underwent 353 sedations for an average of 4.8 sedations/patient (74% increase). Ninety-one (26%) were DS (333% increase). Medication combinations were similar as above for DS (90%) and MS (93%). Nineteen percent of patients had an ASA of 3 or greater.

Our hospitalist performed 16% of the sedations in the BPB timeframe and 72% in the HB timeframe. In the BPB group there were 4 desaturation events (0.02%) among all sedations, compared to 8 desaturation and 1 hemodynamic change events (0.025%) in the HB group. Total work relative value units (wRVUs) and anesthesia units of service (for the DS) generated were 599.6 and 133 respectively in the BPB group compared to 1104.2 (84% increase) and 784 (489% increase) respectively in the HB group. During the BPB timeframe 40 patients were taken to the OR for dressing changes for a total of 69.3 hours, compared to zero in the HB timeframe.

**Conclusions:**

Transitioning from a BPB to HB sedation service resulted in increased sedation numbers and percentage of patients undergoing a sedation, especially DS, as well as increased RVU generation. Sedations were performed in patients with higher ASA scores without a significant increase in adverse events. There was operating room cost and time savings. HB sedation services can be an efficacious and safe model in a burn center.

